# Severe Pseudomonas aeruginosa Pneumonia in a Breast Cancer Patient Despite Pegfilgrastim Administration

**DOI:** 10.7759/cureus.57156

**Published:** 2024-03-29

**Authors:** Yuna Fukuma, Tsunehisa Nomura, Tsuyoshi Mikami, Katsuhiro Tanaka, Naruto Taira

**Affiliations:** 1 Breast and Thyroid Surgery, Kawasaki Medical School, Okayama, JPN

**Keywords:** pegfilgrastim, pseudomonas aeruginosa, febrile neutropenia, chemotherapy, breast cancer

## Abstract

Pegfilgrastim dramatically reduces febrile neutropenia (FN) caused by high-risk chemotherapy. This report details the presentation of a 72-year-old female who developed a fatal infection of Pseudomonas aeruginosa pneumonia that occurred during preoperative chemotherapy despite pegfilgrastim administration. She was brought to the hospital with symptoms of high fever and general fatigue during chemotherapy, but her respiratory symptoms were minimal, and a chest computed tomography (CT) showed no obvious signs of pneumonia. She had FN. After she was hospitalized, her breathing and consciousness worsened rapidly, and the chest CT showed prominent lobar pneumonia. Her blood cultures suggested P. aeruginosa, so she was quickly switched to meropenem. She was diagnosed with septic shock and acute respiratory distress syndrome due to severe P. aeruginosa pneumonia, and she was started on noninvasive positive pressure ventilation with immunoglobulin preparations. P. aeruginosa developed drug resistance, so it was necessary to change antibiotics. She was discharged without complications of pulmonary fibrosis on chest CT. It is crucial to always be aware that severe infections can occur even with pegfilgrastim administration, promptly identify the causative pathogen, and intervene with early treatment.

## Introduction

Pegfilgrastim was approved and made available as a long-acting granulocyte-colony stimulating factor (G-CSF) agent in 2014 [1]. Pegfilgrastim reduced the duration and incidence of neutropenia and the risk of febrile neutropenia (FN) and made high-risk regimens such as dose-dense therapy safe and easy to implement [2].

The American Society of Clinical Oncology (ASCO) guidelines recommend the prophylactic use of G-CSFs when the risk of FN exceeds 20% [3].

FN may occur infrequently, but caution is still required. Pseudomonas aeruginosa pneumonia is a severe infection that is usually fatal and generally occurs in neutropenic patients [4]. Mortality is as high as 28-44% even with appropriate empirical treatment [5,6]. Despite the use of pegfilgrastim, there are few reports of fatal P. aeruginosa pneumonia in cancer patients undergoing neoadjuvant chemotherapy.

## Case presentation

The patient was a 72-year-old woman who had undergone breast-conserving surgery for left breast cancer 17 years earlier. The histological diagnosis was estrogen receptor-positive invasive ductal carcinoma. She was treated with an aromatase inhibitor and followed up regularly at our hospital. Although no abnormalities were observed five months earlier, she was aware of a left breast mass and visited our hospital. A 1.7 cm mass was palpated in the upper inner quadrant of the left breast. The left axillary lymph node was not palpable. A needle biopsy showed low-sensitive hormone receptors (estrogen receptor 30% and progesterone receptor 0% positive) and Her2-negative. The Ki67 labeling index was 48.6%. Because of the rapid expansion of the lesion, after consultation with the patient, EC therapy (epirubicin, cyclophosphamide) with pegfilgrastim was selected as preoperative chemotherapy. However, on day 10 of first-line chemotherapy, the patient presented with high fever, nausea, and general malaise, and she was admitted to the emergency department. On admission, the patient had impaired consciousness (Japan coma scale I-2), temperature of 39.5 °C, pulse of 105/min, respiratory rate of 21/min, blood pressure of 137/70 mmHg, and SpO2 of 95% (room air). Auscultation of the heart and chest was normal.

The laboratory data on admission are shown in Table 1.

**Table 1:** The laboratory data on admission

Lab parameter	Patient values	Reference range
White blood cells (/μL)	190	3,300-8,600
Neutrophils (/μL)	40	1,720-6,880
Lymphocytes (/μL)	150	660-3,440
Red blood cells (×10^4^ /μL)	377	386-492
Hemoglobin (g/dL)	12.3	11.6-14.8
Platelets (×10^4^ /μL)	11.5	15.8-34.8
Blood urea nitrogen (mg/dL)	12	8-20
Creatinine (mg/dL)	0.6	0.46-0.79
Total bilirubin (mg/dL)	0.7	0.4-1.5
C-reactive protein (mg/dL)	7.37	0.0-0.14
β-D glucan (pg/mL)	<6.0	0.0-11.0
PT-INR	1.01	0.88-1.12
APTT (s)	33	26.9-38.1
D-dimer (μg/mL)	1.3	<1.0
Arterial blood gas (room air)		
pH	7.48	7.35-7.45
paO_2 _(mmHg)	67.9	80-100
pCO_2_ (mmHg)	34.9	35-45
HCO_3_^-^ (mmol/L)	25.8	22-26
Base excess (mmol/L)	2.7	╴2.0 to 2.0
Bacterial and viral test		
Urinary legionella pneumonia	(-)	(-)
Aspergillus antigen	(-)	(-)
Influenzae A	(-)	(-)
Influenzae B	(-)	(-)

**Figure 1:**
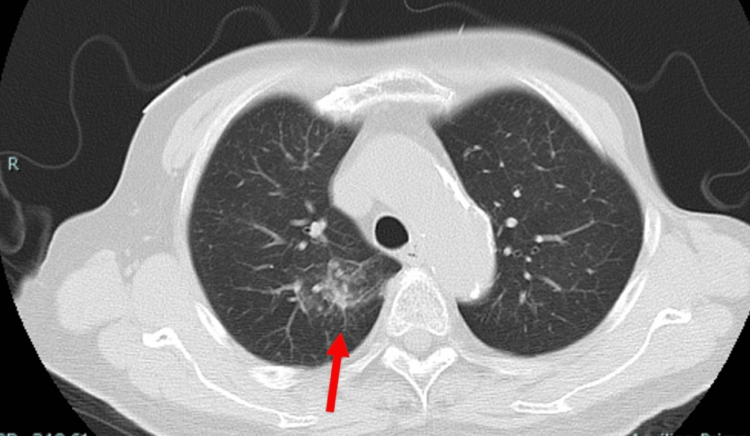
Chest CT at the hospitalization revealed scattered cloudy shadows on the right lobe (red arrow)

She had severe neutropenia and an inflammatory response. Arterial blood gas analysis showed hypoxemia. CT of the chest showed scattered frosted shadows in both upper lobes, a finding that was suspicious for pneumonia, but lacking an obvious source (Figure 1).

**Figure 2:**
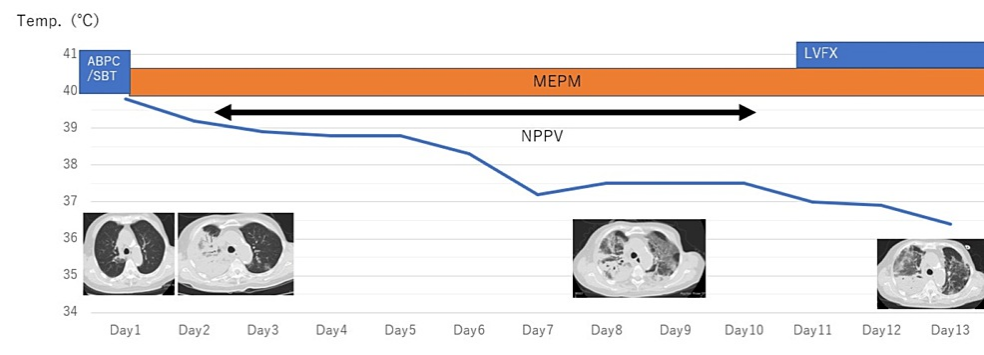
Body temperature, CT change, and antibiotic change are shown (between admission to day 13) ABPC/SBT: sulbactam/ampicillin, MEPM: meropenem, LVFX: levofloxacin

During her hospitalization, she was diagnosed as having FN with suspected pneumonia and a DIC score of 2 and 3 systemic inflammatory response syndrome (SIRS) diagnostic criteria (temperature of > 38 °C, heart rate of > 90/min, respiratory rate of > 20/min). After blood culture, the patient was initially treated with intravenous sulbactam/ampicillin. However, the high fever persisted, respiratory and consciousness status deteriorated rapidly, and blood pressure decreased (Figure 2).

Blood culture showed Gram-negative bacilli, and P. aeruginosa infection should be considered, so the antimicrobial agent was changed to meropenem (Table 2).

**Table 2:** Blood and sputum culture test a: hospitalization, b: 19 days after hospitalization

a: Antibiotic sensitivity testing		
Blood culture (hospitalization)		
P. aeruginosa	MIC (µg/mL)	Interpretation
Piperacillin	<8	S
Cefepime	<2	S
imipenem	<2	S
Meropenem	<2	S
Gentamicin	<2	S
Tobramycin	<2	S
Levofloxacin	<0.5	S
Ciprofloxacin	<0.25	S
Piperacillin/Tazobactam	<8	S
b: Antibiotic sensitivity testing		
Sputum culture (19 days after hospitalization)		
P. aeruginosa	MIC (µg/mL)	Interpretation
Piperacillin	16	S
Cefepime	16	I
imipenem	>8	R
Meropenem	>8	R
Gentamicin	8	I
Tobramycin	<2	S
Levofloxacin	>4	R
Ciprofloxacin	>2	R
Piperacillin/Tazobactam	16	S

**Figure 3:**
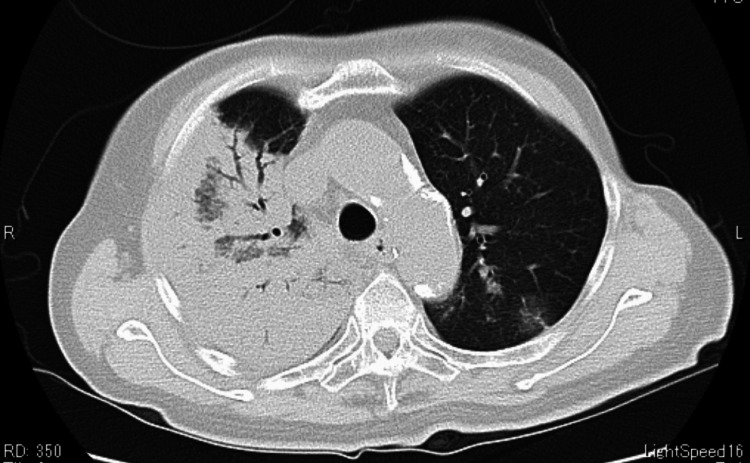
Chest CT performed 48 hours after admission showed severe pneumonia in the right lobe

CT of the chest 48 hours after admission showed significant lobar pneumonia (Figure 3).

**Figure 4:**
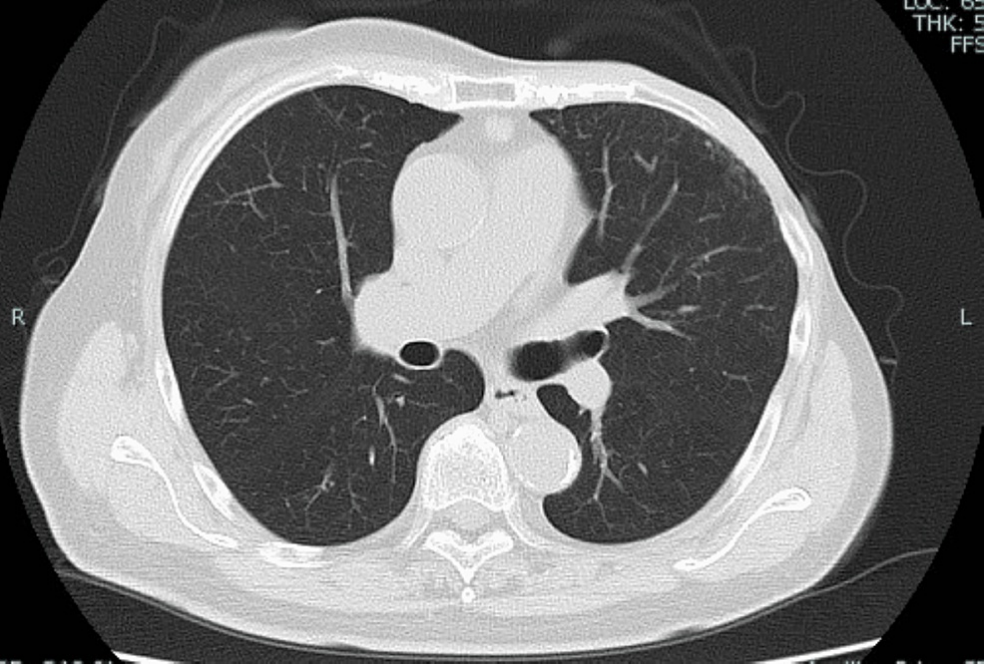
Chest CT (six months after onset) It showed improvement without pulmonary fibrosis.

An immunoglobulin preparation was also administered for severe infection. Her level of consciousness worsened further, and she also had a sepsis-induced hypotension. She was diagnosed with concurrent septic shock and acute respiratory distress syndrome and required non-invasive positive pressure ventilation. Thereafter, her respiratory condition gradually improved, and her fever tended to decrease, but the patient continued to have low-grade fever, so levofloxacin was added. Due to persistent low-grade fever, blood and sputum culture tests performed again detected P. aeruginosa resistant to meropenem and levofloxacin (Table 2), so levofloxacin was changed to ceftazidime plus tobramycin. After the antibiotic change, the patient’s condition improved, and she was discharged ambulatory on day 37 after admission. After discharge, she was followed-up regularly, and chest CT showed improvement without pulmonary fibrosis (Figure 4).

## Discussion

Neutropenia associated with breast cancer chemotherapy is one of the relatively frequent adverse events, and FN is a serious complication that can be life-threatening due to the increased risk of infection. According to the American Society of Clinical Oncology and the European Society for Medical Oncology, when using high-risk regimens (>20% incidence of FN), pegfilgrastim is recommended not only to reduce the incidence of FN but also for its cost-effectiveness through reduced antibiotic use and hospitalization costs [7,8].

The incidence of FN in breast cancer patients before pegfilgrastim approval was reported to be approximately 8.5% with 5FU + epirubicin + cyclophosphamide therapy (FEC) [9,10]. Although less than 10% in Europe and North America, FN develops in about 20% of similar regimens in Japan [9-11]. In a Japanese phase III trial of pegfilgrastim for breast cancer patients, the incidence of FN in the group without pegfilgrastim in perioperative docetaxel and cyclophosphamide therapy was as high as 68.8%, whereas the incidence of FN in the treated group was 1.2% [12]. In dose-dense therapy, G-CSF was administered as primary prevention in all cases, the FN incidence rate was 2%, and the grade 4 neutropenia incidence rate was considerably suppressed at 9%.

When FN develops, it is very important to determine whether it will become severe. Klastersky proposed a method of identifying patients with a score of 21 or higher as a low-risk group using the Multinational Association for Supportive Care in Cancer System (MASCC) score [13]. Although the incidence of severe infections was reported to be less than 5% when evaluated as a low-risk group with this score, the present case had a score of 19 points and was classified as high risk [14].

In high-risk cases, cefepime, meropenem, tazobactam/piperacillin, ceftazidime, etc., which are β-lactam drugs with anti-P. aeruginosa activity, should be administered intravenously, and drugs should be changed according to the results of susceptibility testing. P. aeruginosa is an attenuated bacterium that rarely causes infections in healthy individuals, but it is important as a causative agent of opportunistic infections in immunocompromised hosts. Mortality is as high as 28-44% even with appropriate empirical treatment [5,6]. Similar to the present case, a neutropenic patient with minimal respiratory symptoms, other than fever at initial presentation, rapidly developed (within 48 hours) acutely progressive pneumonia with bacteremia [15]. Factors associated with a poor prognosis in P. aeruginosa infection include pneumonia, septic shock, persistent and severe neutropenia, and delay in initiation of antibiotic therapy [16]. Cases presenting with pneumonia or septic shock have a high mortality rate. In 127 cases of P. aeruginosa pneumonia among patients with hematologic malignancies, we reported a mortality rate of greater than 80% if appropriate treatment was not started early, and 21% if treatment was started without delay [17].

The resistance mechanism of P. aeruginosa is thought to involve four factors: (1) biofilm formation, (2) inhibition of antimicrobial agent entry into and exit from the bacteria, (3) mutation of the site of action of antimicrobial agents, and (4) enzymes that degrade antimicrobial agents. P. aeruginosa has biological characteristics that make it difficult for drugs to penetrate the body due to the biofilm formed by mucoids. It also acquires resistance to carbapenems such as imipenem by regulating the protein synthesis that makes up the pump-out system, called the multi-efflux pump system; this is a biological feature of the disease. It has also been reported that the continued presence of antimicrobial agents in the growth environment of P. aeruginosa causes genetic mutations in the site of action, and strains that acquire drug resistance survive and become resistant to penicillin antibiotics such as ampicillin and cephalosporins through the production of cephalosporinase dependent on the AmpC gene [17]. In the present case, it was necessary to change to ceftazidime plus tobramycin because P. aeruginosa is resistant to meropenem and levofloxacin was detected on repeat culture.

Thus, P. aeruginosa pneumonia is a serious complication in patients with neutropenia during chemotherapy. Appropriate therapeutic intervention can be implemented early by identifying and conducting drug susceptibility testing. There was only one case of severe P. aeruginosa pneumonia despite the use of pegfilgrastim [18]. Although pegfilgrastim administration has now dramatically reduced the frequency of FN and made drug management easier than before, we must not forget that it can be severe.

## Conclusions

Pegfilgrastim dramatically reduces febrile neutropenia caused by high-risk chemotherapy. In the present case, despite the absence of underlying conditions in a breast cancer patient, a severe infection of P. aeruginosa pneumonia occurred during preoperative chemotherapy with pegfilgrastim administration.
